# An Inordinate Fondness of Rarity

**DOI:** 10.1371/journal.pbio.1001573

**Published:** 2013-05-28

**Authors:** Jonathan Chase

**Affiliations:** Freelance Science Writer, Saint Louis, Missouri, United States of America

In evolutionary biologist circles, J.B.S. Haldane's quip to a theologian that from his studies, he has learned God has an “inordinate fondness of beetles”, is a perennial favorite to quote, misquote and modify, even though it's unclear whether the encounter ever actually happened. The point was simply that, but for the vagaries of evolution, beetles were among the most diverse groups of species on the planet. What is unsaid, however, is that the vast majority of species in this very diverse group are also very rare, often known from only a single location or specimen. A rather universal pattern in any given community, be it beetles, trees, or bacteria, is that a few species are quite common, but the majority are rare, many exceptionally so. In fact, when we compare the hyperdiverse tropics to the more depauperate temperate zone, the primary difference in diversity occurs among the rarest of the species. So, while God may be fond of beetles, he seems to abhor dominance for most of his creation.

**Figure:**
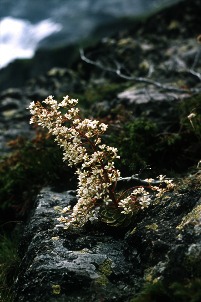
Photo of the spectacular ***Pyramidal Saxifrage*** **(** ***Saxifraga cotyledon*** **), a plant that may provide distinctive and vulnerable functions to alpine ecosystems thanks to unusual combinations of traits such as exceptionally long flowering stems, making it an important resource for pollinators.** Photo Credit: J.P. Dalmas.

Humans appear to be the cause of the impending sixth mass extinction of life on Earth (the fifth was most likely at the hands of an asteroid crashing to Earth 65 million years ago, causing the extinction of the dinosaurs and around 75% of other plant and animal species). As awareness of this impending biodiversity crisis has grown, a burgeoning literature examining the relationship between biodiversity and the functions of ecosystems (e.g., biomass production, nutrient cycling) has emerged, typically showing a positive relationship. The value of biodiversity, it is surmised, is that the functioning of entire ecosystems would collapse if the impending biodiversity crisis is not curtailed.

Unfortunately, there has always been a nagging hole in the logic placed on justifying the conservation of biodiversity in order to maintain the functioning of ecosystems. If our only reason for protecting species is to preserve the functions of ecosystems, many conservation biologists worry, perhaps it is just fine to let the rare majority of species to go extinct. Implicitly assumed here is that because rare species are, well, rare, they do not play a particularly strong role in the functioning of ecosystem. Further, rare species are typically assumed to be “redundant” with respect to their functions, especially in speciose systems, such that their loss would have minimal influence on the ecosystem. This assumption that rare species are redundant, however, is loaded with ambiguity, and in reality, very little is known about how common and rare species differ (or do not differ) in their functional traits and thus their possible roles in the ecosystem. That is, until now.

In this issue of *PLOS Biology*, Mouillot and colleagues set out to determine whether rare species are indeed functionally redundant within ecosystems, or instead, whether they possess unique traits that might have disproportionate ecosystem-level effects if they went extinct. To do so, they amassed an impressive dataset combining large-scale surveys of species' commonness and rarity with information on their functional traits from three very different ecosystems—846 coral reef fishes from the South Pacific, 2,979 species of alpine plants from the French Alps, and 662 species of tropical trees from French Guiana. The scientists defined rare species as those that represented less than 5% of the local abundance or regional occupancy of the most common species and categorized species according to their functional traits using a multivariate measure that essentially asks, “how distinct are a species' traits from others in the group?” In alpine plants, for example, several species of grasses are quite similar in their traits, whereas more distinct species provide more unique functions, such as *Saxifraga cotyledon*, which provides important floral resources for pollinators and *Cytisus polytrichus*, which produces seeds that provision its seed-dispersing ants. They compared these distinctiveness values to the rarity of the species, finding that those that were most distinct were also among the rarest. Next, they estimated an index of functional vulnerability of each species, calculated by comparing the similarity of a species' traits to other members of the community and weighting that by the other species' relative commonness. If a species has traits similar to a very common species, it does not support much functional vulnerability and its loss would not strongly influence on the functioning of the ecosystem. Overall, they found that common species tended to be redundant with other common species, whereas rare species tended to support the most vulnerable functions in an ecosystem.

Importantly, Mouillot and colleagues recognized an important limitation of these initial analyses— chance. Because there are many more rare than common species in any given community, correlations between rarity and functional vulnerability could emerge as a result of simple probability. To control for this, Mouillot and colleagues developed a null model where they randomly assigned species into vulnerability categories and compared this expectation to the vulnerability supported by rare species that they observed. On the whole, they found that rare species supported vulnerable functions much more than expected by chance, indicating the unique functional role that a large proportion of rare species seem to play among these highly disparate types of ecosystems.

Although speculative, one consequence of these results is that rare species with unique functions might help buffer the response of ecosystems to global change. For example, coral reefs are vulnerable to overgrowth by macroalgae in the face of many global changes. On Australia's Great Barrier Reef, common but functionally redundant parrotfishes (Labridae) and rabbitfishes (Siganidae) are unable to control macroalgae blooms, while the functionally distinct, but rare, batfish (*Platax pinnatus*) plays a keystone role in restricting macroalgae and maintaining coral reefs. Other rare species, waiting in the wings, might provide similarly important ecosystem functions today and in the future.

**David Mouillot M, Bellwood DR, Baraloto C, Chave J, Galzin R, et al. (2013) Rare Species Support Vulnerable Functions in High-Diversity Ecosystems. doi:10.1371/journal.pbio.1001569**

